# Assessing Pubic Symphysis Evolution in Guinea Pigs (*Cavia procellus*): Insights From Computer Tomography on Primiparous and Non‐Breeding Females

**DOI:** 10.1002/vms3.70076

**Published:** 2024-10-22

**Authors:** Sabrina Vieu, Héloïse Hugon, Samuel Boucher, Clément Bercker, Jean‐François Bruyas, Marion Fusellier

**Affiliations:** ^1^ Service des Nouveaux Animaux de Compagnie, Oniris, CHUV Nantes France; ^2^ Oniris, INRAE, BIOEPAR Nantes France; ^3^ Service de Reproduction, Oniris, CHUV Nantes France; ^4^ Service Transversal d'Imagerie Médicale, Oniris, CHUV Nantes France; ^5^ Labovet Conseil, BP539 Les Herbiers France; ^6^ Oniris, Nantes Université, Inserm, RMeS Nantes France

**Keywords:** computed tomography, guinea pig, symphysis

## Abstract

In guinea pigs (*Cavia porcellus*), dystocia is a common occurrence. Several factors have been identified in the literature, including the ossification of the pubic symphysis following failure to breed before 9–12 months of age. The objective of this study was to investigate the ossification of pubic symphysis and its evolution during growth in two groups of females. The first group consisted of non‐breeding females, while the second group comprised females introduced to breeding at 4–6 months of age. Twelve pairs of sows were selected for comparison, with one non‐breeding and one breeding sow in each pair. Symphysis width and tissue density were assessed using micro‐computed tomography. Measurements included the distance between the acetabula, width and bone density of the pubic symphysis. Serial computed tomography scans were performed on each sow over several months, both before and after parturition. The results revealed a significantly higher symphysis width in females that had bred. In addition, symphysis ossification was absent in both breeding and non‐breeding sows, contrary to previous descriptions of this species. Therefore, dystocia in guinea pigs may not be attributable to ossification of the pubic symphysis.

## Graphical abstract

In guinea pigs, dystocia is common and occasionally linked to ossification of the pubic symphysis. This study compared symphysis width and bone density in breeding and non‐breeding female guinea pigs using micro‐computed tomography. The results revealed no ossification in either the pregnant or non‐pregnant groups, but bred females exhibited a significantly wider pubic symphysis.

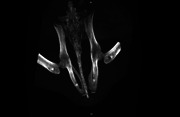

## 1 Introduction

Guinea pigs (*Cavia porcellus*) are popular pets and valued experimental models due to their biological similarities to humans (Cameron, Holder, and Connor 2022; Cohen, Kwok, and Huang 2020; Gao et al. 2018; Pritchett Corning, Mulder, and Henderson 2018). However, they experience parturition issues more frequently compared to other rodents and rabbits.

Dystocia, a common complication, can result in stillbirth, pup mortality and sow mortality. Signs of dystocia include continuous straining for 20 min or the presence of bloody or green discharge without pup expulsion, as well as unproductive contractions lasting over 2 h. In addition, pressure from the gravid uterus or pups can cause temporary paresis or paralysis of the pelvic limbs. In cases where medical treatment fails, a caesarean section may be necessary, though the prognosis remains guarded to poor (Kondert and Mayer 2017; Pignon and Mayer 2021).

To understand and reduce the incidence of dystocia in guinea pigs, several hypotheses have been proposed. One primary cause suggested is the inadequate separation of the pubic symphysis (Czarnecki and Adamski 2016; Martinho 2006; Pignon and Mayer 2021). This issue is exacerbated by the relatively large size of the guinea pig foetus in relation to the mother, considering the gestation period of 65–71 days, which results in offspring that are almost self‐sufficient at birth (Kondert and Mayer 2017; Weir 1974). Consequently, a narrowed pelvic canal may significantly contribute to dystocia. Other contributing factors include obesity, uterine torsion, uterine atony and vitamin C deficiency (Bishop 2002; Pignon and Mayer 2021).

The estimated lifespan of guinea pigs in captivity is 5–7 years. A controversial theory suggests that females who do not breed before 8 to 12 months old experience ossification of the fibrocartilage of the pubic symphysis. This ossification could prevent the symphysis from widening during parturition, leading to dystocic births (Bishop 2002; Hoefer and Latney 2009; Kondert and Mayer 2017). However, it has been reported that some females give birth for the first time after 1 year of age without complications (Kondert and Mayer 2017; Pignon and Mayer 2021).

Based on this theory, many breeders opt to breed females at an early age to avoid dystocic births and reduce maternal and neonatal death rates. Despite its prevalence in literature, the origin of this statement is unclear, and there appears to be no formal proof supporting it (Bishop 2002).

Questions regarding bone density, including the ossification of the symphysis, have been frequently discussed in the literature. One method to measure symphysis bone density is through diagnostic imaging. Computed tomography (CT) can provide measurements of mineral bone density using Hounsfield units (HUs), offering valuable insights. CT has gained popularity in veterinary hospitals due to its ability to provide clear images without tissue superimposition and enhanced contrast resolution (Hoefer and Latney 2009). Looking ahead, micron‐scale CT (micro‐CT) presents a promising future perspective. This imaging technology, based on the same principles as clinical CT, offers higher‐resolution imaging capabilities (Vilaplana Grosso 2019). Initially designed for laboratory animals, micro‐CT shows potential as a diagnostic imaging modality for small species like the guinea pig (average weight 1–1.5 kg) (Clark and Badea 2014; C.‐F. Lee et al. 2010; Souza et al. 2006; Vilaplana Grosso 2019). The process of CT involves the production of x‐rays, which penetrate tissues and undergo an attenuation. The HU is a quantitative scale used to measure the radiodensity of each pixel in the image, derived from the linear attenuation coefficient measurement (Snyder and Schneider 1991; S. Lee et al. 2013). Assessment of bone mineral density using HUs from CT has become a valuable tool in veterinary medicine, offering a non‐invasive means of evaluating bone quality (Buenger et al. 2021; D. Lee et al. 2015; Mejia et al. 2019; Simion et al. 2023).

This study aimed to investigate the evolution of the pubic symphysis in breeding and non‐breeding female guinea pigs by evaluating its width and density using micro‐CT.

## 2 Materials and Methods

### 2.1 Experimental Design and Subject Selection

After several dystocia in his breeding, a veterinarian guinea pig breeder and co‐author (S.B.) was advised to perform a CT examination of the pelvis as part of the clinical work‐up for his guinea pigs. This follow‐up aimed to prevent the reproduction of potentially problematic lines, as bone symphysis issues were suspected as the cause of dystocia.

Sows aged between 3 and 6 months were brought by the owner, with various breeds such as Abyssinian, crowned, agouti, self‐beige, cream and Havana. Priority was given to selecting sisters of the sows. If a sister sow was unavailable, pairing was done with a related female when possible or with an unrelated female of nearly the same age as a last resort. All guinea pigs were identified with coloured spots on their ears for individual recognition. Each pair consisted of one female selected randomly for breeding and the other kept as a control. The control females were allowed to reproduce after the experiment if no pelvic abnormalities were observed on CT examination. Breeding commenced when the females reached the age of 5 and 6 months through natural mating. Following parturition, the breeding female did not undergo reproduction again for the duration of the study. The sows were housed in pairs in outdoor enclosures and provided ad libitum access to hay, vegetables and a measured seed diet formulated for guinea pigs (Bellanné). They also had continuous access to fresh water throughout the study period. All guinea pigs were considered healthy based on their medical history, physical examination and the absence of observable signs of disease, including normal feeding behaviour.

CT scans were conducted at the veterinary teaching hospital between September 2013 and September 2014. According to the literature, pubic symphysis ossification in female guinea pigs typically occurs between 6 and 9 months (Peters 1997). CT scans were therefore performed every 6–8 weeks from 3 to 12 months of age. CT scans were not performed during the last third of pregnancy to avoid potential complications, but they were conducted after birth for breeding females. All CT examinations were conducted under general anaesthesia, administered via intramuscular injections of ketamine (5 mg/kg, Imalgene 1000; Boehringer), butorphanol (1 mg/kg, Torphasol; Axience) and midazolam (1 mg/kg, Midazolam Aguettant 5 mg/mL; Aguettant), with an approximate duration of 20 min. Guinea pig tolerance to CT scans was assessed based on feeding behaviour and general examination post‐anaesthesia.

### 2.2 Data Recording Procedures

All micro‐CT scans were conducted following a standardised protocol. CT and data collection were carried out for all sows under general anaesthesia. The CT scans were performed using a micro‐CT scanner (Siemens Inveon PET/CT scanner, Siemens Medical Solutions) with a voltage of 46 kV and an intensity of 6.4 mAs. The slice thickness was set to 110 µm with a field of view of 100 mm, resulting in a spatial resolution of 110 µm in‐plane and providing an isotropic voxel resolution of 110 µm.

During the scans, the animals were positioned in ventral recumbency with their limbs placed on either side of a carbon‐fibre pallet, which served as a stable platform with minimal attenuation of gamma rays. To prevent overlap of the public symphysis with the caudal vertebrae, a slight rotation was performed. The x‐beam was centred 0.5 cm behind the iliac wings in the median plane. No contrast‐enhanced CT images were acquired during the scans.

Images obtained from the CT scans were recorded and measurements were conducted using electronic callipers with medical image analysis software (OsiriX v6.0.2, Bernex, Switzerland).

**FIGURE 1:**
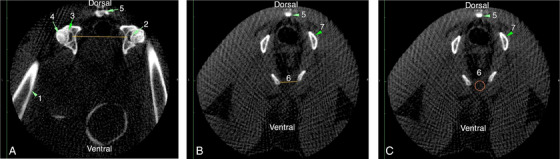
(A) Measurement of inter‐acetabular distance (IAD). (B) Measurement of pubic symphysis width (PSW). (C) Measurement of pubic symphysis density in a breeding female guinea pig. 1, Femur; 2, femoral head ligament insertion; 3, acetabula; 4, femoral head; 5, cross‐section of a vertebra; 6, pubic symphysis; 7, cross‐section of ischium.

To minimise errors associated with operator changes, measurements were performed multiple times on the scanner images for each scan series by a single person. The analysis included the following measurements: (i) inter‐acetabular distance (IAD), defined as the distance between the acetabula at the insertion point of the femoral head ligament, (ii) pubic symphysis width (PSW), defined as the width of the pubic symphysis at its narrowest point and (iii) density within the area of the pubic symphysis at the same location (Figure 1).

### 2.3 Statistical Analysis

In the study, two different parameters were evaluated. First, PSW was compared between breeding and non‐breeding sows. In addition, the IAD‐to‐PSW ratio was calculated. For each CT scan series of each group, the mean and standard deviation of this ratio were determined. The data were assessed for normality, and a Student's *t*‐test for paired series was performed to compare the relationship between PSW and IAD. The null hypothesis stated that no difference existed between the two sets of sows. Significance was considered for *p* < 0.05. Statistical analyses were performed using R software version 3.2.2 (R Foundation for Statistical Computing, Vienna, Austria).

Furthermore, the density of the pubic symphysis was measured based on the Hounsfield scale, which corresponds to the contrast in the obtained images and is directly related to the linear attenuation coefficient. The results were divided into three categories (≤ 0, > 0 and < 300 and ≥ 300). These qualitative data facilitated a comparison between the categories.

## 3 Results

### 3.1 Study Population

A total of 23 sows were included, allowing the formation of 11 pairs consisting of one breeding and one non‐breeding female. Initially, 12 pairs were formed, but one male was mistakenly included at the outset. The remaining female from this pair underwent other CT examinations. On average, each guinea pig underwent three to five CT examinations. Anaesthesia was generally well tolerated by most sows, with the exception of four sows that exhibited movement during anaesthesia, including one during a CT examination.

Some measurements could not be carried out for the following reasons: pregnancy or mating (11 measures), death (10 measures), tardive inclusion (4 measures), animal movement during acquisition (1 measure), sexing error (1 measure) and omission (3 measures). Images acquired from the mistakenly pregnant female were excluded from the statistical analysis of pubic symphysis measurements but were included for density measurement analysis.

Following the deaths of two sisters initially associated with dystocia for the pregnant sow of Pair 3 and uterine haemorrhage for Pair 4, adjustments were made. A new pair was formed between two half‐sisters sharing the same father but not from the same litter. In addition, another pair was established comprising unrelated individuals to ensure reliable statistical analysis. Furthermore, a mistake occurred where the non‐pregnant sow of Pair 5 was erroneously brought into reproduction, resulting in pregnancy during the fifth CT scan.

### 3.2 Pubic Symphysis Density

**FIGURE 2:**
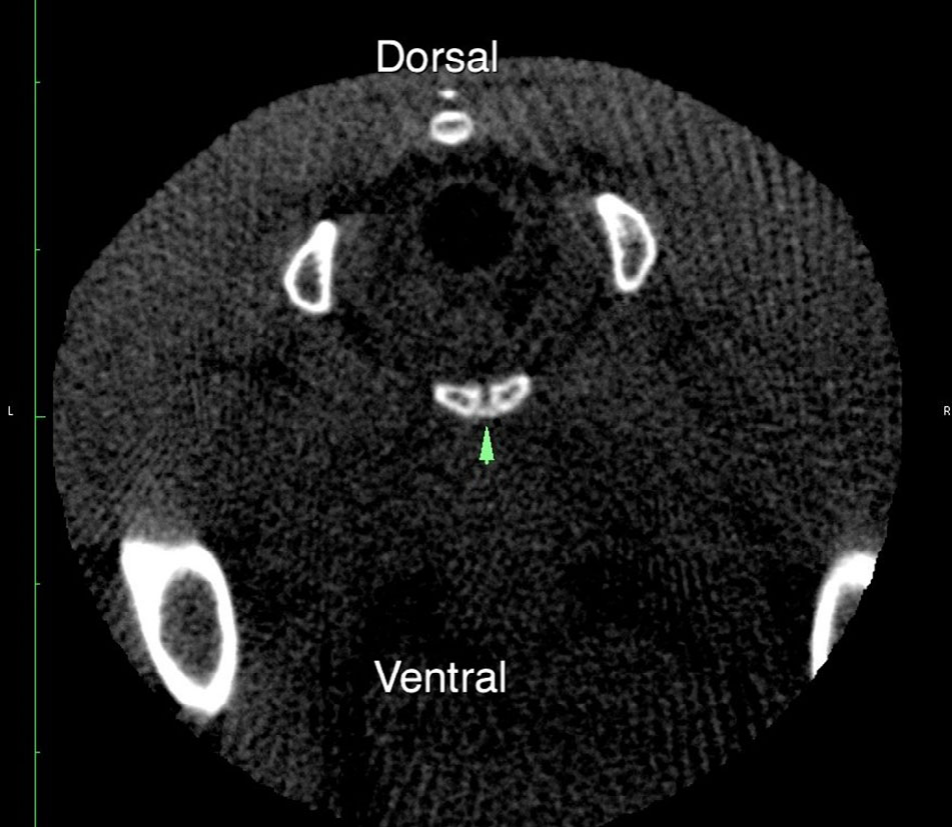
Dense bridle (arrow) connecting the two pubic bones in a male guinea pig of 8 months. Symphysis density was measured at 343 HU.

The results of the bone density measurements are summarised in Table 1. In the initial three series of scans, no clear trend is evident. However, from scan nos. 4 and 5 onwards, a notable homogenisation of density is observed. Negative values, corresponding to air or fatty tissue, are consistently observed in 100% of cases, regardless of whether the female is a breeder or not. Furthermore, once a female exhibits negative density on a scan, it never returns to positive density on subsequent scans. It is worth noting that the male guinea pig, while not included in the statistical analysis, exhibited bone density at the pubic symphysis. This was characterised by the presence of a dense bridle connecting the two pubic bones (Figure 2). Notably, among all sows under 1 year old within our sample that underwent the scans, no densities exceeding 300 units were observed.

**TABLE 1:** Public symphysis density measurements across five CT scans, categorised by Hounsfield unit categories (≤ 0, > 0 and < 300 and ≥ 300), in 23 sows and one male. Ages in months of each animal are provided in corresponding columns of Hounsfield categories.

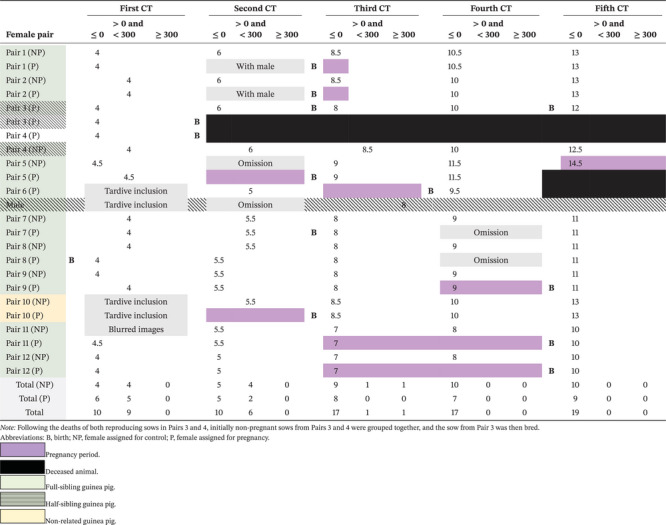

### 3.3 Measurement of the Pubic Symphysis

The distance between the two pubic bones and the distance between the two acetabula were initially measured, and then the ratio was calculated. The results are summarised in Table 2. Among the 23 guinea pig sows, 8 pairs (*n* = 16) met the criteria for the first scanning session, 6 pairs (*n* = 12) were included in the second scanning session, 8 pairs (*n* = 16) for the third, 6 pairs (*n* = 12) for the fourth and 9 pairs (*n* = 18) for the fifth session. Other pairs were excluded due to the events listed above.

**TABLE 2:** Ratio of IAD‐PSW for five CT scans in 21 sows.

		Ratio on CT 1		Ratio on CT 2		Ratio on CT 3		Ratio on CT 4		Ratio on CT 5
Pair 1 (NP)		5.88		1.677		1.879		1.842		2.182
Pair 1 (P)		1.63		x		x	**B**	12.445		12.62
Pair 2 (NP)		2.21		2.319		1.665		2.208		2.518
Pair 2 (P)		2.327		x		x	**B**	12.108		13.341
Pair 4 (NP)		2.414		1.842		2.035		1.858		1.88
Pair 4 (P)		2.143		1.603	**B**	1.813		28.21	**B**	8.263
Pair 5 (NP)		1.585		x		1.744		0.964		24.807
Pair 5 (P)		2.058		x	**B**	32.917		14.769		
Pair 6 (P)		x		1.554		X	**B**	33.372		
Pair 7 (NP)		1.501		1.677		1.364		1.378		1.028
Pair 7 (P)		0.995		1.681	**B**	5.615		x		30.748
Pair 8 (NP)		1.461		1.818		1.151		2.442		1.709
Pair 8 (P)	**B**	1.742		1.114		1.569		x		11.87
Pair 9 (NP)		2.537		5.74		1.523		0.74		3.795
Pair 9 (P)		1.92		4.256		1.471		2.056	**B**	3.367
Pair 10 (NP)		x		2.259		1.641		1.789		1.301
Pair 10 (P)		x		x	**B**	38.786		21.446		24.326
Pair 11 (NP)		x		1.151		3.294		2.06		5.347
Pair 11 (P)		x		1.448		13.966		x	**B**	8.537
Pair 12 (NP)		1.74		1.224		1.233		4.398		9.45
Pair 12 (P)		1.057		1.231		16.269		x	**B**	13.991
Mean ± SD (NP)		2.416 ± 1.367		2.19 ± 1.309		1.753 ± 0.577		1.968 ± 0.955		3.245 ± 2.534
				
Mean ± SD (P)	**1**	1.734 ± 0.457	**1**	1.889 ± 1.077	**5**	12.66 ± 13.53	**8**	17.77 ± 9.873	**12**	14.118 ± 7.956
Sum of each birth between each CT	**1**		**1**		**5**		**8**		**12**	

Abbreviations: B, birth; NP, female assigned for control; P, female assigned for pregnancy.

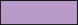
Pregnancy period.

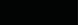
Deceased animal.

Given the erroneous inclusion of the non‐pregnant sow from Pair 5 in the reproductive group during the fifth CT, it was excluded from the subsequent Student's *t*‐test analysis for the last CT session. Following the birth of at least five sows, the mean IAD‐to‐PSW ratio increased in the pregnant sow group, and this ratio remained higher after birth. A Student's *t*‐test was conducted on the means of non‐breeding and breeding sows for each session, yielding *p* values of 0.24, 0.23, 0.05, 0.012 and 0.09 for Sessions 1, 2, 3, 4 and 5, respectively. Consequently, the null hypothesis was rejected for Scan Sessions 3–5, indicating a significant difference in mean PSW between non‐breeding and breeding sows during these sessions.

**FIGURE 3:**
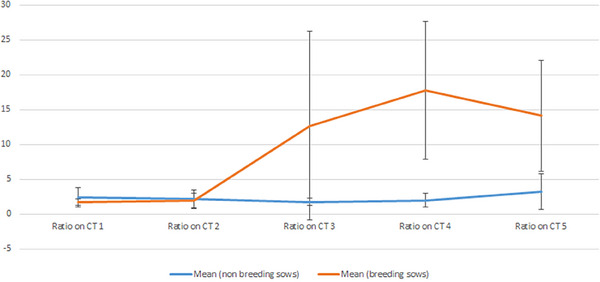
Distribution of mean pubic symphysis width means evolution between breeding and non‐breeding female guinea pigs during Scanner Sessions 1–5.

Moreover, a comparison of the mean IAD‐to‐PSW ratio before and after parturition, that is, between Scanners 1 and 5 (Figure 3), revealed a significant increase (*p* = 0.012) after parturition. An increase in symphysis ratio of a similar magnitude to that observed in younger reproductive females was noted in the females that were erroneously bred late at the age of 14 months (Pair 5—CT 5: 24.807).

No significant difference was found in the mean IAD‐to‐PSW ratio between Sessions 1 and 5 for non‐breeding females (*p* = 0.62). However, the *p* value obtained for breeding females was 0.012, indicating a significant increase in the size and width of the pubic symphysis ratio after parturition. Identical comparisons were performed for non‐breeding females on the same scanning sessions (Scan Sessions 1 and 5), yielding a *p* value of 0.62. This result suggests that there is no significant change in the symphysis ratio mean in non‐breeding females.

## 4 Discussion

This study investigates the changes in guinea pig pubic symphysis among breeding and non‐breeding sows using multiple micro‐CT measurement techniques, focusing on variations between pre‐ and post‐parturition conditions. Detailed measurements of the width and bone density of the pubic symphysis in guinea pigs before and after birth, compared to non‐breeding sows, were conducted in 23 sows predominantly related. The follow‐up micro‐CT examinations allowed us to refute the hypothesis of symphysis ossification before 12 months of age. In addition, our findings indicate that the symphysis width in breeding females does not significantly differ from that of nulliparous females in our sample. However, it is observed to enlarge after parturition based on our data.

Diagnostic imaging was selected for its convenience, and this technology has been utilised for years to study dystocia and parturition in both humans and animals (Koca, Yilmaz, and Avcilar 2023; Koné et al. 2022; Rajaonarison Ny Ony Narindra et al. 2018). Initially, abdominal radiography was considered due to its routine use in veterinary practice. However, accurate measurements would have been challenging on radiographs due to the small size of guinea pigs and the superimposition of the symphysis with the pelvic channel in two‐dimensional imaging techniques. A study investigating the pelvic cavity of rabbits demonstrated that CT enables accurate three‐dimensional evaluation and measurements of the pelvis (Özkadif, Eken, and Kalaycı 2014; Van Caelenberg et al. 2011). Micro‐CT was ultimately chosen for its excellent precision in investigating the parameters of interest and its minimally invasive nature. High‐resolution images provided by micro‐CT allow for various measurements and reconstructions, offering more detail compared to classic CT or radiography, particularly in small species (Capello, Lennox and Ghisleni 2015; Souza et al. 2006; Van Caelenberg et al. 2011).

Various guinea pig breeds were included in the study. While guinea pigs generally exhibit slight differences in size between breeds, the satin breed is reported to have bone fragility, making it an interesting addition to the study (Gallego 2017; Jordan et al. 2009).

Our sample size may introduce bias and be influenced by the availability of sister sows, chosen to ensure a more reliable comparison. A comparison between different guinea pig breeds and origins could have provided valuable insights with a larger sample size.

Although the anaesthetic protocol was generally well‐tolerated, three animals exhibited movement while asleep and one during image acquisition, suggesting that deeper anaesthesia might have improved image quality. Gas anaesthesia with isoflurane or sevoflurane is recommended for its safety and relative ease of application to achieve Stage 3 anaesthesia (Bennett and Lewis 2022). However, it was not feasible due to space limitations in the micro‐CT device. Initially, CT scans were planned every month, but to mitigate anaesthetic risks and owner availability, they were conducted every 6–8 weeks. This interval also accounts for the guinea pig gestation period of 59–72 or 65–71 days, according to different sources (Kondert and Mayer 2017; Weir 1974).

In the field of theriogenology, pelvimetry is commonly used to measure the pelvis and can be valuable for investigating dystocia (Dobak et al. 2018; Tsousis et al. 2010; Vernooij et al. 2020). Various measurements are typically obtained through this method, including the width and length of the pelvis, pelvic inlet and caudal pelvic aperture. Despite the availability of several measurements in other species, there is a limited description of pelvimetry in guinea pigs. In our study, we primarily focused on questioning the symphysis for its enlargement and modification. This is why we chose to concentrate solely on this parameter. However, an analysis of pelvic dimensions might be beneficial for further research involving dystocia in this species.

Considering that the experiment involved growing animals whose size varied throughout the protocol, it was important to account for this factor. To address this, a ratio of the distance between both pubic bones and the distance between both acetabula of each individual was calculated. Measuring the distance between the acetabula at the site of insertion of the femoral head ligament provided a reliable reference point for repeat measurements and was not dependent on the positioning of the animal.

The statistical tests indicate no difference between the mean symphysis width of breeding and non‐breeding sows at the first scan. This suggests that random selection created two homogeneous groups, which had no influence on the results regarding PSW after parturition for breeding females or at the last scan for non‐breeding females. In addition, the standard deviation in non‐breeding females shows minimal variations within this group.

It is noteworthy that the pubic symphysis of breeding sows increases in width, whereas this measurement remains constant in non‐breeding sows. These findings imply that these variations could be attributed to pregnancy, which is supported by studies in humans demonstrating modifications of the pelvis during the perinatal period (Morino et al. 2019).

The symphysis density remained within the range of ≤ 0–300 HU over the 4‐to‐12‐month period in all sows. While there was no observed ossification in either breeding or non‐breeding females, the male guinea pig mistakenly included exhibited bone density with a dense bridle at the pubic symphysis compared to the females. Several studies in other species suggest that reproductive history and sex both influence bone microarchitecture (de Bakker et al. 2018; Nahkur et al. 2017). Given the suspected differences between sexes, a comparison with a group of males could be informative (Bonjour et al. 1991; Naganathan and Sambrook 2003). To confirm our results, bone biopsy or bone microstructural analyses could be beneficial. A study involving virgin females, freshly euthanised primiparous guinea pig sows and intact males details symphysis composition in young American short‐haired guinea pigs 12–15 weeks old (Ortega et al. 2003). In females, the inter‐pubic joint is formed of fibrocartilage (a true symphysis), with bones joined at term by a connective ligament, constituting a syndesmosis. In contrast, males have a hyaline cartilage joint. These findings can explain the variations observed in our results regarding symphysis density. Modifications of the pubic joint depend on the age, sex and physiological reproductive stage studied (Ortega et al. 2003).

Having a single observer perform all measurements could potentially introduce bias. To mitigate this effect, measures were repeated several times to verify consistency and avoid measurement errors. While the accuracy of measurements could have been evaluated by involving both inexperienced and experienced observers (Vernooij et al. 2020), it is important to note that the size of the animals studied and the measurements being in centimetres with millimetre variations may have minimised the potential for observer variability. It is worth mentioning that other studies with several observers are typically conducted on humans and large animals, where larger differences in measurements may occur (Korhonen et al. 2010; Vernooij et al. 2020).

Furthermore, other limitations of this study are inherently linked to the fact that not all animals received the same number of scans and sow ages varied from one CT to another. Although guinea pigs reach full weight in less than a year, it would have been interesting to perform scans on older animals and monitor symphysis width evolution after parturition. It is reported that guinea pig skeletons continue to evolve after one year of age (Witkowska et al. 2014). To enhance our results, comparisons could be made with guinea pigs of different ages.

To investigate the causes of dystocia in guinea pigs, diagnostic imaging could be a valuable tool to prevent fetopelvic disproportion. A combined method utilising both foetal and maternal factors could be considered. In cattle, for example, the ratio of the foetal hoof width to the longitudinal diameter of the maternal pelvis before calving is used to estimate the risk of dystocia (Hiew et al. 2016; Maeda, Kitahara and Osawa 2022).

In conclusion, our results reveal that the pubic symphysis of guinea pigs does not undergo ossification during the first year or after parturition, even in the absence of early gestation.

Thus, the symphysis remains fibrocartilaginous, allowing for parturition beyond growth.

We observed a significant difference in symphysis size between reproductive and non‐reproductive females, as well as a difference between measurements taken before and after the first birth. These findings suggest that the pubic symphysis undergoes modifications related to reproductive events, highlighting its importance in understanding dystocia in guinea pigs.

## Author Contributions

Héloïse Hugon, Samuel Boucher, Jean‐François Bruyas led the conceptualisation and collected the data. Héloïse Hugon, Clément Bercker and Sabrina Vieu analysed the data. Jean‐François Bruyas and Marion Fusellier supervised the project and supported investigation. Sabrina Vieu drafted the paper. Sabrina Vieu, Marion Fusellier and Clément Bercker reviewed the manuscript.

## Ethics Statement

The clinical follow‐up was conducted without causing harm or cruelty to the animals and was overseen by the veterinarian, who was also the breeder of the animals. All procedures were performed under the supervision of veterinary personnel.

## Conflicts of Interest

The authors declare no conflicts of interest.

### Peer Review

The peer review history for this article is available at https://publons.com/publon/10.1002/vms3.70076.

## Data Availability

The data presented in this study are available on request from the corresponding author.
